# The case of a 75‐year‐old male with confusion and acute blindness

**DOI:** 10.1002/acn3.51658

**Published:** 2022-10-07

**Authors:** Ryan Donaghy, Brian Stamm, Fan Z. Caprio

**Affiliations:** ^1^ Department of Neurology Northwestern University Feinberg School of Medicine Chicago Illinois USA

## Summary of Case

A 75‐year‐old man with no known past medical history presented with acute‐onset binocular vision loss and anterograde amnesia. Several hours prior to their presentation to the hospital, he was found in his car after being seen driving erratically and having driven off the road into the grass. On presentation to the emergency department, the patient remained disoriented. During the interview, the patient reported that he was unable to see any of his surroundings in either eye. He could not tell when he had been moved from a darker room to a room with bright lights. Examination showed atrial fibrillation, absent blink‐to‐threat bilaterally, disorientation, bilateral intact pupillary light responses, and largely intact motor and sensory functions. Laboratory data revealed hypercholesteremia and pre‐diabetes. CT angiography and subsequent conventional angiogram revealed bilateral, proximal posterior cerebral artery (PCA) occlusions.

**Figure 1:**
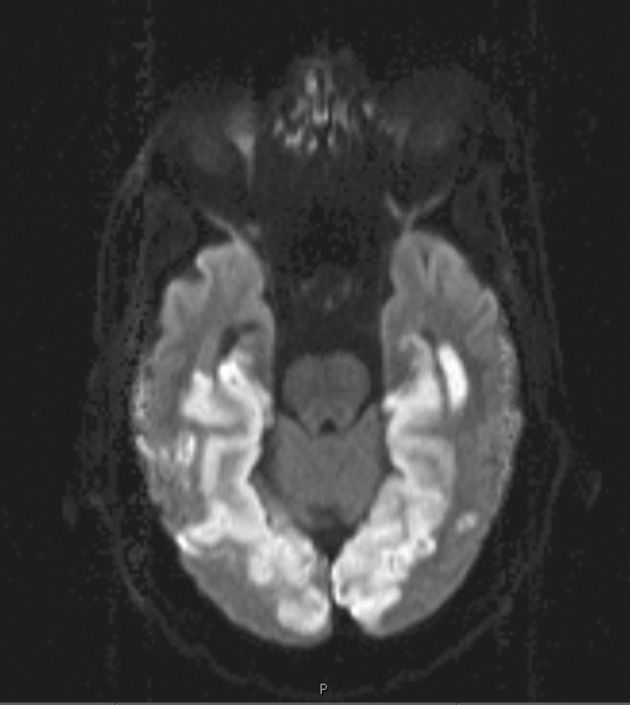
MRI Brain, diffusion‐weighted imaging. These representative axial MRI diffusion‐weighted images reveal areas of diffusion restriction in the bilateral PCA distributions (medial temporal lobes, occipital lobes).

The patient proceeded emergently to the interventional angiography suite and underwent successful catheter‐based retrieval of both P1 clots resulting in TICI‐3 reperfusion. Subsequent brain MRI showed acute multifocal infarcts involving medial temporal and occipital lobes, in bilateral PCA territories. His intact pupillary light reflex in the setting of persistent vision loss suggested cortical blindness. After the thrombectomy, he had episodes of confabulation about regaining sight, consistent with Anton syndrome.^1^ He was initially managed with antiplatelet therapy in the setting of his large infarct burden post‐thrombectomy and was ultimately transitioned to a direct oral anticoagulant for secondary stroke prevention in the setting of depressed cardiac ejection fraction and persistent atrial fibrillation. Prompt recognition of visual deficits in acute stroke patients remains a diagnostic challenge. Visual field deficits are underrepresented in stroke screening tools, which can result in the under‐identification of such deficits.^2^ Bilateral PCA occlusion with bilateral thrombectomy is exceedingly rare but remains an important stroke syndrome that can be effectively intervened upon if recognized emergently (Figure 1).

## Diagnosis

Bilateral Proximal PCA Territory Ischemic Stroke.

## Take‐Home Points

1. Acute stroke management requires rapid clinical reasoning and decision making.
2. Posterior circulation syndromes are underrepresented on standardized stroke evaluations but must remain on an examiner's differential of acute neurologic syndromes.
3. Anton Syndrome consists of cortical blindness and visual anosognosia, often evidenced by confabulation about retaining sight.
